# Entwicklung gewaltassoziierter penetrierender Traumata in der Metropolregion Düsseldorf über einen fünfjährigen Zeitraum (GewPen-Studie)

**DOI:** 10.1007/s00101-024-01420-6

**Published:** 2024-05-22

**Authors:** Jutta Schürmann, Mark Michael, Olaf Picker, Dan Bieler, Kalle Heitkötter, Thomas Tremmel, Bernd Schnäbelin, Michael Bernhard

**Affiliations:** 1 Zentrale Notaufnahme, Universitätsklinikum Düsseldorf, Heinrich-Heine-Universität, Moorenstraße 5, 40225 Düsseldorf, Deutschland; 2 Klinik für Anästhesiologie, Universitätsklinikum Düsseldorf, Heinrich-Heine-Universität, Düsseldorf, Deutschland; 3 Klinik für Orthopädie und Unfallchirurgie, Universitätsklinikum Düsseldorf, Heinrich-Heine-Universität, Düsseldorf, Deutschland; 4 Klinik für Unfallchirurgie und Orthopädie, Wiederherstellungs- und Handchirurgie, Verbrennungsmedizin, Bundeswehrzentralkrankenhaus Koblenz, Koblenz, Deutschland; 5 Gesundheitsamt Düsseldorf, Düsseldorf, Deutschland; 6 Feuerwehr der Stadt Düsseldorf, Düsseldorf, Deutschland; 7 Referat für Rettungswesen und Gesundheitlicher Bevölkerungsschutz, Ministerium für Arbeit, Gesundheit und Soziales des Landes Nordrhein-Westfalen, Düsseldorf, Deutschland

**Keywords:** Gewalt, Städtisches Einsatzgebiet, Transsektorale Versorgung, Notaufnahme, Violance, Urban setting, Transsectoral care, Emergency department

## Abstract

**Einleitung:**

Penetrierende Verletzungen sind eine seltene, aber immer wieder vorkommende Einsatzsituation in der Notfallmedizin. Das Ziel der Untersuchung war es, die Häufigkeit und Verletzungscharakteristika penetrierender, gewaltassoziierter Verletzungen einer Metropolregion über einen 5‑jährigen Zeitraum zu ermitteln.

**Material und Methodik:**

In der retrospektiven Untersuchung wurden anhand einer Datenbankabfrage der Leitstelle des Rettungsdienstbereichs Düsseldorf sämtliche rettungsdienstliche Einsätze mit penetrierenden, gewaltassoziierten Verletzungen in den Jahrgängen 2015, 2017 und 2019 identifiziert und deskriptiv analysiert. Für diejenigen Patienten, die dem überregionalen Traumazentrum (ÜTZ) zuverlegt wurden, erfolgte neben der präklinischen eine weitergehende transsektorale Analyse des innerklinischen Verlaufes.

**Ergebnisse:**

In den 3 Jahrgängen 2015, 2017 und 2019 konnten insgesamt 266 Patienten (Alter: 33 ± 14 Jahre, männlich: 79 %) mit penetrierenden, gewaltassoziierten Verletzungen erfasst werden (2015 vs. 2017 vs. 2019: *n* = 81 vs. *n* = 93 vs. *n* = 92). Die am häufigsten betroffene Altersgruppe war zwischen 15 und 34 Jahre alt. Eine höhere Einsatzhäufigkeit fand sich für die Stadtbezirke Altstadt, Stadtmitte und einen weiteren Stadtteil (Oberbilk). Eine hohe Einsatzhäufigkeit fand sich in den Nächten von Samstag auf Sonntag zwischen 20.00 und 04.00 Uhr. Rettungsdiensteinsätze mit Notarztbeteiligung nahmen über die Jahrgänge zu (2015 vs. 2019: 27 vs. 42 %, *p* = 0,04). Als Tatwaffe kamen vorwiegend Messer (56 %), abgeschlagene Glasflaschen (18 %) und Scherben (6 %) zum Einsatz. Im ÜTZ kamen 71 aller Patienten (27 %, Injury Severity Score 11 ± 14) zur Aufnahme. Bei diesen Patienten stiegen über die Jahre der Anteil einer unmittelbar erfolgten operativen Versorgung (2015 vs. 2019: 20 vs. 35 %, *p* < 0,05) und ein positiver Alkoholnachweis an (2015 vs. 2019: 10 vs. 43 %, *p* < 0,05). Die 30-Tages-Letalität betrug 1,1 % (*n* = 3).

**Schlussfolgerung:**

Penetrierende, gewaltassoziierte Verletzungen sind relevante, aber seltene rettungsdienstliche und innerklinische Einsatzsituationen. Zukünftige Versorgungsstrategien sollten sich auf die Stationierung von Rettungskräften in Schwerpunkteinsatzbereichen („Altstadtwache“, Hauptbahnhof) und Präventionsstrategien auf Waffen- und Glas‑/Flaschenzonen ausrichten. Eine Steuerung des Alkoholkonsums sollte diskutiert werden.

**Zusatzmaterial online:**

Zusätzliche Informationen sind in der Online-Version dieses Artikels (10.1007/s00101-024-01420-6) enthalten.

## Abstract (en)

**Introduction:**

Penetrating injuries are a rare but recurring emergency situation in the out-of-hospital and in-hospital emergency settings. The purpose of this study was to determine the incidence and characteristics of injuries associated with penetrating violence across a German metropolitan region over a 5-year period.

**Material and methods:**

In the retrospective study, a database query of the control center of the Düsseldorf rescue service area was used to identify and descriptively analyze all rescue service operations with penetrating violence-associated injuries in the years 2015, 2017, and 2019. For those patients who were transferred to the major trauma center, a further analysis of the in-hospital course was performed.

**Results:**

In the 3 years 2015, 2017 and 2019 a total of 266 patients (age: 33 ± 14 years, male: 79%) could be recorded (2015 vs. 2017 vs. 2019: *n* = 81 vs. *n* = 93 vs. *n* = 92, respectively). The most common age group involved had an age range of 15–34 years. A particularly higher frequency of emergency calls was found for the areas of Old Town, City Center, and one other district (Oberbilk). A high frequency of rescue missions was found in the nights from Saturday to Sunday between 20.00 p.m. and 04.00 a.m. Rescue missions with emergency physicians on board increased over the years (2015 vs. 2019: 27 vs. 42%, *p* = 0.04). The primary weapons used were knives (56%), broken glass bottles (18%) and broken glasses (6%). Out of all patients 71 (27%, injury severity score 11 ± 14) were admitted to the major trauma center. Among these patients, the proportion of immediate surgical care (2015 vs. 2019: 20% vs. 35%, *p* < 0.05) and positive alcohol detection increased over the years (2015 vs. 2019: 10% vs. 43%, *p* < 0.05). The 30-day mortality in the 3 years studied was 1.1% (*n* = 3).

**Conclusion:**

Penetrating injuries associated with violence are relevant but rare rescue missions. Future care strategies should focus on deployment of rescue resources close to the scene of the incident (“old town guard”, central station), and prevention strategies should focus on weapon prohibition zones. A control of alcohol consumption should be discussed.

## Einleitung

In den letzten Jahren nahm die Zahl der Körperverletzungsdelikte in der Landeshauptstadt Düsseldorf, insgesamt betrachtet, leicht ab (2013: *n* = 5526, 2018: *n* = 5168; −6,5 %), gleichzeitig nahmen aber die Fälle, die als gefährliche und schwere Körperverletzung definiert wurden, zu (2013: *n* = 1463, 2018: *n* = 1582; +8,1 %) [24]. Im Gegensatz zu den Zahlen der vorgenannten *Polizeilichen Kriminalitätsstatistik* beschreiben die ermittelten Zahlen des TraumaRegister der Deutschen Gesellschaft für Unfallchirurgie (DGU®) über eine Dekade einen tendenziell leichten Rückgang penetrierender Traumata und eine Zunahme stumpfer Traumata (stumpf vs. penetrierend 2018: 96,2 vs. 3,8 %; 2009: 95,2 vs. 4,8 %) [7]. Aufgrund von Biaseffekten der Grundgesamtheit, beispielweise durch eine höhere Einschlussrate auch minderschwerer Fälle, könnte diese Veränderung in den Ergebnissen des TraumaRegister DGU® über die Jahre aber allein durch die Zunahme leichter Fälle mit stumpfen Traumata oder aber weiterer Selektionskriterien begründet sein.

Traumata werden in stumpfe und penetrierende Traumata eingeteilt. Stumpfe Gewalt wird dabei definiert als eine mechanische Einwirkung einer stumpfkantigen Fläche gegen den menschlichen Körper [1]. Gewaltassoziierte penetrierende Traumata umfassen Verletzungen, bei denen mit einem spitz-/scharfkantigen Werkzeug oder einer Waffe die Haut und evtl. auch tiefer liegende Strukturen verletzt wurden (Penetration von lat. penetrare „eindringen, durchdringen“). Hierzu zählen neben Stich- auch Schnittverletzungen durch entsprechende Waffen und waffenähnliche Objekte.

In der prähospitalen Einsatzrealität, in der innerklinischen Versorgung und auch in der tagtäglichen medialen Berichterstattung wirkt es so, als ob sowohl die Anzahl als auch die Schwere der gewaltassoziierten penetrierenden Verletzungen ständig zunehme. Möglicherweise wird diese Wahrnehmung aber durch die intensive mediale Beschäftigung mit dem Thema „Terror“ und „Gewalt“ beeinflusst [11, 13, 15, 26, 27]. Vor diesem Hintergrund werden Studien benötigt, die sich mit der Klärung der Entwicklung gewaltassoziierter penetrierender Traumata beschäftigen.

Ziel der vorliegenden Studie war zu untersuchen, ob über die Jahrgänge 2015, 2017 und 2019 Änderungen der Häufigkeit, der Verletzungsschwere und der Einsatzschwerpunkte gewaltassoziierter penetrierender Traumata in der Metropolregion der Stadt Düsseldorf auftraten.

## Material und Methodik

### Studiendesign

In der retrospektiven GewPen-Studie (*Gew*alt-assoziierter *pen*etrierender Traumata in der Metropolregion Düsseldorf) wurden in Form einer anonymisierten Kohortenstudie alle Patienten, die vom Rettungs- und Notarztdienst der Landeshauptstadt Düsseldorf in den Jahrgängen 2015, 2017 und 2019 wegen einer gewaltassoziierten penetrierenden Verletzung behandelt wurden, eingeschlossen. Die Studie wurde durch die Ethikkommission der Medizinischen Fakultät der Heinrich-Heine-Universität genehmigt (Nr. 2020-1019).

### Prähospitale Datenerfassung

Routinemäßig und unabhängig von der Studie wird durch den Rettungs- und Notarztdienst bei allen Patienten eine einsatzbegleitende verpflichtende schriftliche digitale Dokumentation mittels NIDA-Pad (Fa. MedDV GmbH, Stadt Fernwald, Deutschland) durchgeführt, und die digitalen Daten werden nach Einsatzabschluss auf einem Server der Landeshauptstadt Düsseldorf gespeichert.

Die Einsatzdaten der zu untersuchenden Jahrgänge wurden durch die Fa. MedDV in Excel (Microsoft Excel® für MAC, Microsoft, Redmond, VA, USA, Version 16.78) exportiert. Im nächsten Schritt wurden die Dateien in einer Excel-basierten Datenbank aggregiert und alle nichtrelevanten Datenfelder entfernt, um die Performance der weiteren Datenabfrage zu optimieren, eine Anonymisierung der Rohdatendatei durch den Ärztlichen Leiter Rettungsdienst durchgeführt und anschließend die Datenbank makrogestützt nach vorab definierten Schlüsselwörtern als Ein- und Ausschlusskriterien durchsucht (Tab. 1).

**Tab. 1:** Ein- und Ausschlusskriterien für die Identifikation von Rettungs- und Notarzteinsätzen mit „penetrierenden Verletzungen“

*Ausschlusskriterien*		
Wespe	Biene	Insekt
*Einschlusskriterien*		
Stich	Stilett	Flinte
Stiech^a^	Schwert	Bolzen
Schuuss^a^	Machete	Polizei
Schuß	Glasflasche	KV
Schuss	Scherbe	Schnittverl
Penetrierend	Streit	Schlägerei
Messerstech^a^	Schlägerei	Perfora
Pistole	Pfählung	Pneumothorax
Revolver	Körperverletzung	Flasche
Waffe	Dolch	Stecherei
Gewehr	Schwert	–

*KV* Körperverletzung

^a^Einsatzstichwörter weisen intendiert eine inkorrekte Rechtschreibung auf, um diese Einsätze in der Stichwortsuche in den Rettungsdienstprotokollen identifizieren zu können

Alle automatisch selektierten Datensätze wurden anschließend noch einmal händisch validiert und dabei Fälle von Selbstverletzungen, suizidalen Handlungen und durch stumpfe Gewalt verursachte Wunden (z. B. Bretter, Baseballschläger, Hammer, intakte Glasflaschen) ausgeschlossen.

Einsatzprotokolle von Rettungswagen und Notarzteinsatzfahrzeugen (NEF) des gleichen Einsatzes wurden händisch identifiziert und zu einem Gesamtdatensatz aggregiert. Doppelungen der rettungsdienstlichen Einsätze konnten so ausgeschlossen werden. Den identifizierten Einsätzen mit penetrierenden gewaltassoziierten Verletzungen wurden die Einsatzzeiten und die Global-Positioning-System(GPS-)Daten aus dem zugehörigen Protokoll des Einsatzleitrechners der Leitstelle Düsseldorf zugeordnet und in der Datenbank ergänzt. Aus den Einsatzprotokollen wurden die studienrelevanten Parameter in die Datenbank extrahiert (z. B. Alter, Geschlecht, Verletzungsschwere nach NACA-Score, Tatwaffe, prähospitale Versorgung und Versorgungszeiten, Zieldestination).

### Innerklinische Datenerfassung

Im transsektoralen Ansatz wurden der innerklinische Verlauf der dem überregionalen Traumazentrum (ÜTZ) zugeführten Patienten analysiert. Hierzu wurden die innerklinischen Daten (z. B. Verletzungsschwere gemäß Injury Severity Score, ISS) aus dem Schockraumprotokoll, Entlassbericht, Operationsberichte und die Dokumentation der Zentralen Notaufnahme aus dem Patientendatenmanagementsystem(PDMS)-System (COPRA, COPRA System GmbH, Berlin, Deutschland) mit den Daten des Krankenhausinformationssystem (KIS, MEDICO, Fa. Cerner Deutschland GmbH, Idstein, Deutschland) evaluiert und in der Datenbank ergänzt. Die Datenbank wurde vor der weiteren deskriptiven Analyse wieder anonymisiert. Ein Rückschluss auf den individuellen Fall war aus diesen Daten nicht mehr möglich. Somit wurden die Anforderungen an den Datenschutz nach der Datenschutz-Grundverordnung (DSGVO) eingehalten.

### Statistik

Zur statistischen Analyse wurden definierte Patientenkollektive bezüglich diverser Merkmale miteinander verglichen. Alle Daten wurden mittels Excel ausgewertet und die Abbildungen mittels DataGraph (Version 4.6.1, Fa. Visual Data Tool Inc., Chapel Hill, NC, USA) erstellt. Für qualitative Merkmale werden absolute und relative Häufigkeiten in Prozent angegeben. Quantitative Variablen werden als Mittelwert ± Standardabweichung (SD) präsentiert. Außerdem wurden der Median, das Minimum, das Maximum und der Interquartilabstand ermittelt. Unterschiede zwischen Patientenkollektiven bezüglich qualitativer Faktoren wurden mittels eines Chi^2^-Tests untersucht. Bei quantitativen, annähernd normalverteilten Merkmalen kam der *t*-Test für zwei unverbundene Stichproben zum Einsatz. Bei schief verteilten Variablen wurde stattdessen der U‑Test von Mann und Whitney durchgeführt. Ein Testergebnis mit *p* < 0,05 wurde als statistisch signifikant gewertet.

## Ergebnisse

**Abb. 1:**
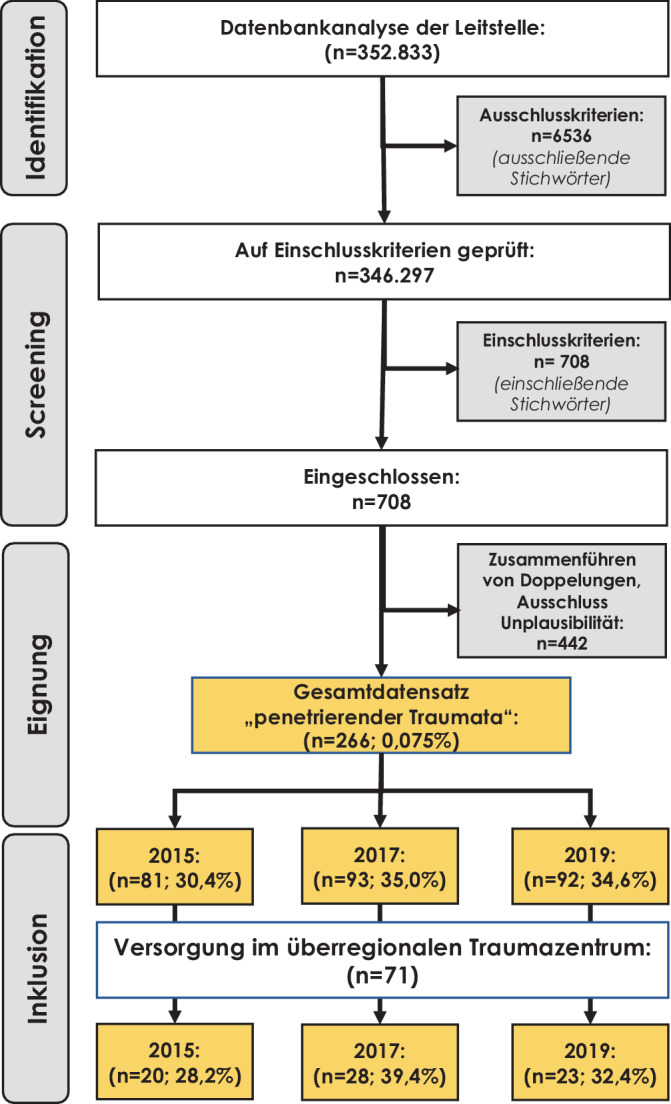
PRISMA-Flow-Diagramm: Ausgehend von einer Datenbankanalyse der Leitstelle wurden penetrierende gewaltassoziierte Verletzungen der Jahre 2015, 2017 und 2019 im Rettungsdienstbereich Düsseldorf identifiziert

In den 3 evaluierten Jahrgängen wurden insgesamt 352.833 Datensätze aus NIDA extrahiert (Abb. 1). Nach Prüfung der Ausschlusskriterien wurden 6536 Datensätze extrahiert. Die in der Datenbank verbliebenden Datensätze wurde hinsichtlich der Einschlusskriterien gescreent und 708 Datensätze identifiziert. Nach Prüfung auf Doppelungen und Plausibilität konnten 266 Datensätze extrahiert werden, die das Studienkollektiv darstellten. Dabei entfielen auf die Jahre 2015, 2017 und 2019 eine vergleichbare Anzahl an Einsätzen (*n* = 81 (30,4 %), *n* = 93 (35,0 %), *n* = 92 (34,6 %)) und insgesamt < 1 % aller Einstätze.

### Demografische Daten

Männliche Patienten waren in dem Patientenkollektiv deutlich häufiger als weibliche Patienten (*n* = 211 (79,3 %) vs. *n* = 55 (20,7 %), *p* < 0,05) vertreten. Das durchschnittliche Alter der Patienten betrug 33 ± 14 Jahre (Median: 30, min-max: 8–80, Interquartilabstand (IQA): 22,5 bis 41 Jahre). Der größte Anteil entfiel auf die Altersgruppe 15 bis 34 Jahre (*n* = 166, 62,4 %), gefolgt von der Altersgruppe 35 bis 74 Jahre (*n* = 95, 35,7 %). Nur 3 Patienten (1,1 %) waren ≤ 14 Jahre und nur 2 Patienten (0,8 %) ≥ 75 Jahre alt.

### Geodatenanalyse

**Abb. 2:**
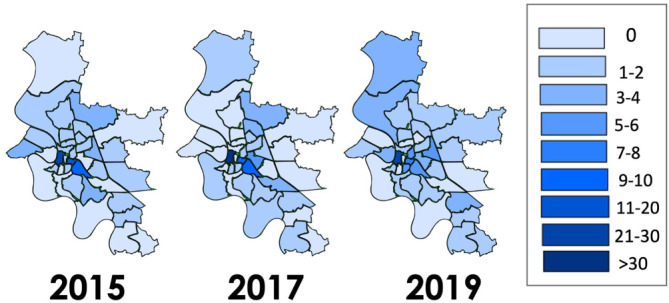
Einsatzlokalisation der 266 Rettungsdiensteinsätze mit penetrierenden gewaltassoziierten Verletzungen im Rettungsdienstbereich der Landeshauptstadt Düsseldorf, zusammengefasst pro Postleitzahlgebiet in den Jahren 2015, 2017 und 2019. Die jeweilige Einsatzhäufigkeit wurde dabei farbig differenziert

Die Analyse der GPS-Daten der Einsatzorte zeigte eine deutliche Häufung der Einsätze in bestimmten Postleitzahlen- und Stadtgebieten (Abb. 2): Führende Ereignislokalisation war die Düsseldorfer Altstadt (*n* = 76, 28,6 %), gefolgt von der Düsseldorfer Stadtmitte (*n* = 47, 17,7 %) und dem Stadtteil Oberbilk (*n* = 25, 9,3 %).

### Einsatztaktische Kennzahlen

Die meisten Einsätze (42,9 %) fanden am Wochenende (Samstag und Sonntag) statt. Hinsichtlich der Einsatzuhrzeiten fanden 55,3 % der Einsätze abends bzw. nachts zwischen 20.00 und 4.00 Uhr statt. Eine Auswertung nach Zeitfenstern und Wochentagen zeigte, dass 73 von 266 Einsätzen (27,4 %) in der Nacht von Samstag auf Sonntag in der Zeit von 20.00 bis 04.00 Uhr stattfanden. Der Anteil an Einsätzen, in denen ein NEF primär oder als Nachforderung zum Einsatz kam, stieg über die Jahre kontinuierlich an (2015 vs. 2017 vs. 2019: *n* = 22 (27,2 %) vs. *n* = 41 (44,1 %) vs. *n* = 39 (42,4 %), 2015 vs. 2017 *p* = 0,02, 2015 vs. 2019 *p* = 0,04).

### Tatwaffen

**Abb. 3:**
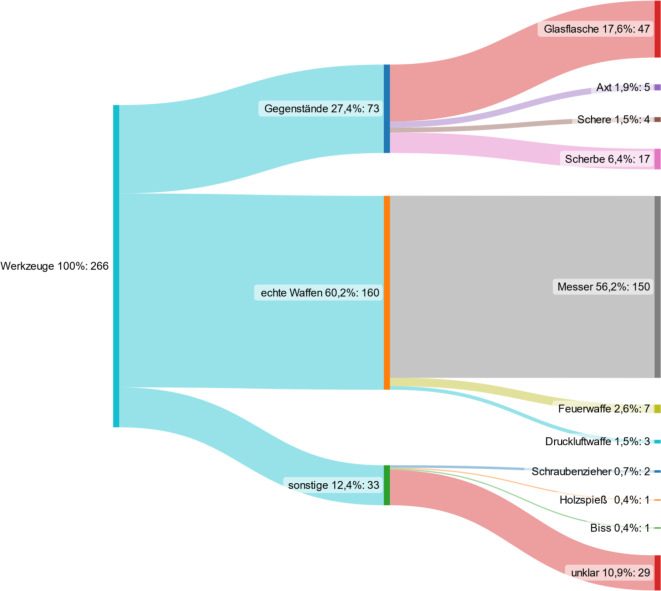
Differenzierung der Tatwaffen bzw. -gegenstände bei den insgesamt 266 Patienten mit penetrierenden gewaltassoziierten Verletzungen im Untersuchungszeitraum

Der Hauptteil der penetrierenden Verletzungen wurden mit einem Messer verursacht (*n* = 150, 56,4 %), gefolgt von abgeschlagenen Glasflaschen (*n* = 47, 17,7 %) und Scherben (*n* = 17; 6,4 %) (Tab. 2**, **Abb. 3). Penetrierende Verletzungen durch Schusswaffen lagen in 7 von 266 Fällen vor (2,6 %). Im Jahr 2017 wurde im Rahmen eines Amoklaufs 5‑mal eine Axt als Tatwaffe eingesetzt.

**Tab. 2:** Übersicht der die penetrierenden Verletzungen verursachenden Tatwaffen

	2015 (*n* = 82) (%)	2017 (*n* = 93) (%)	2019 (*n* = 92) (%)	2015–2019 (*n* = 266) (%)
*Hieb und Stichwaffen*				
Messer	52 (63,1)	50 (53,8)	48 (52,2)	150 (56,2)
Glasflasche	15 (18,3)	17 (18,3)	15 (16,3)	47 (17,6)
Scherbe	3 (3,7)	6 (6,5)	8 (8,7)	17 (6,4)
Axt	–	5 (5,4)	–	5 (1,9)
Schere	–	2 (2,2)	2 (2,2)	4 (1,5)
Schraubenzieher	–	–	2 (2,2)	2 (0,7)
Holzspieß	–	–	1 (1,1)	1 (0,4)
*Schusswaffe*				
Feuerwaffe	3 (3,7)	3 (3,2)	1 (1,1)	7 (2,6)
Druckluftpistole	1 (1,2)	1 (1,1)	2 (2,2)	4 (1,5)
*Sonstige*				
Biss	–	1 (1,1)	–	1 (0,4)
Unklar	8 (9,8)	8 (8,6)	13 (14,1)	29 (10,9)

### Verletzungsschwere

Seitens des Rettungs- und Notarztdienstes wurden in mehr als der Hälfte der Fälle eine geringe Verletzungsschwere gemäß NACA 1 (*n* = 13, 4,9 %) oder 2 (*n* = 152, 57,1 %) angegeben. Mäßige bis schwere, aber nicht lebensbedrohliche Verletzungen mit NACA 3 wurde in 16,5 % (*n* = 44) und eine nichtauszuschließende akute Lebensgefahr (NACA 4) in 13,5 % (*n* = 36) und eine akute Lebensgefahr (NACA 5) in 6,3 % (*n* = 17) konstatiert. Die NACA-Kategorien unterschieden sich in Abhängigkeit vom Transport mittels Rettungswagen bzw. Rettungswagen plus Notarzteinsatzfahrzeug nicht voneinander. Der Anteil der Verletzungsschwere gemäß NACA 4 und 5 stieg über die Jahre nicht an (2015 vs. 2017 vs. 2019: *n* = 13 (16,0 %) vs. *n* = 16 (17,2 %) vs. *n* = 24 (26,1 %)). Zwei Patienten (0,76 %) waren mit NACA 6 reanimationspflichtig am Einsatzort, wovon ein Patient innerklinisch verstarb, und 2 Patienten wurden bereits tot aufgefunden (NACA 7). Die 30-Tages-Letalität betrug 1,1 % (*n* = 3).

### Verletzungslokalisation

**Abb. 4:**
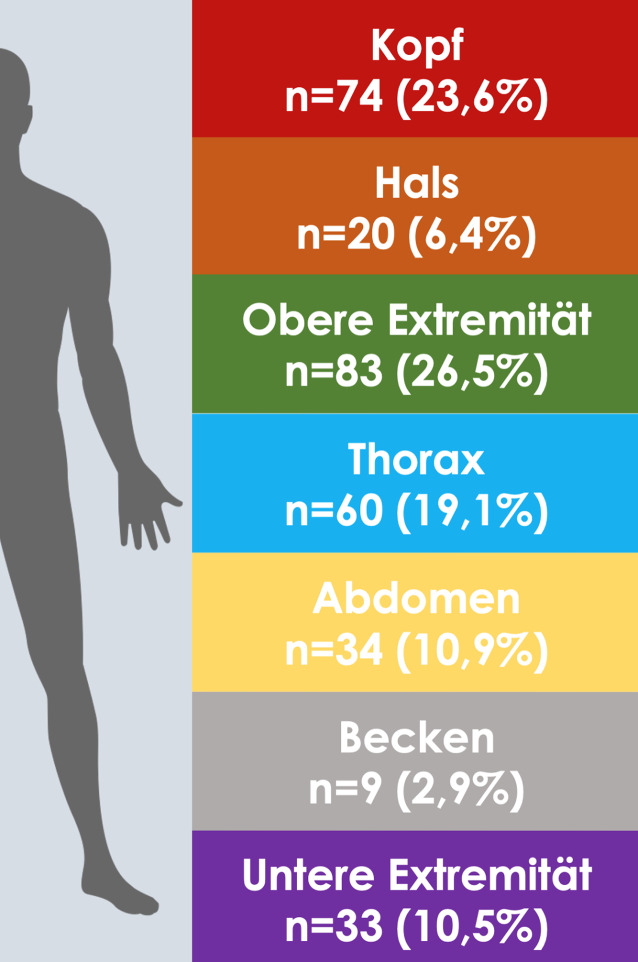
Darstellung der betroffenen/verletzten Körperregionen der untersuchten Patienten (*n* = 266). Hierbei wurden unter Berücksichtigung von Mehrfachverletzungen insgesamt 313 Einzelverletzungen erfasst

Am häufigsten waren die obere Extremität (*n* = 83, 31,2 %) und der Kopf (*n* = 74, 27,8 %) betroffen (Abb. 4). Bei 60 Patienten (22,5 %) wurden Thorax-, bei 34 Patienten (12,8 %) Abdomen- und in 20 Fällen (7,5 %) Halsverletzungen beschrieben. Bei 43 Patienten (16,2 %) wurden Verletzungen von mehr als einer Körperregion im Rettungsdienst- bzw. Notarztprotokoll dokumentiert.

### Rettungsdienstliche und notärztliche Maßnahmen

Bei 4 Patienten erfolgte die prähospitale Atemwegssicherung mittels endotrachealer Intubation. Eine Thoraxdrainage oder Entlastungspunktion wurde bei 2 Patienten durchgeführt (je einmal 2015 und 2017). Einen i.v.-Zugang erhielten 24,8 % aller Patienten, dabei stieg die Rate an periphervenösen Zugängen über die Jahre nicht an (2015 vs. 2017 vs. 2019: *n* = 17 (21,0 %) vs. *n* = 24 (25,8 %) vs. *n* = 25 (27,2 %)). Ein Patient bekam einen i.o.-Zugang. Ein Tourniquet wurde 2017 und 2019 jeweils bei 2 Einsätzen eingesetzt. Tranexamsäure kam im Jahr 2019 bei 4 Patienten zum Einsatz. Ein Chest-Seal-Schnellverband wurde im Rahmen einer thorakalen Messerstichverletzung in 5 Fällen eingesetzt (2017: *n* = 1; 2019 *n* = 4); dreimal wurde im Jahr 2019 Celox® als Hämostyptikum eingesetzt.

### Innerklinische Versorgung

Im transsektoralen Ansatz konnten 71 Fälle des ÜTZ aufgearbeitet werden (2015: *n* = 20 (28,2 %), 2017: *n* = 28 (39,4 %), 2019: *n* = 23 (32,4 %)). Eine Verletzungsschwere gemäß ISS < 9 Punkte („minor trauma“) wiesen 39 Patienten (55,7 %) in den 3 untersuchten Jahrgängen auf. Ein ISS > 25 Punkte („very severe/profound trauma“) fand sich bei 11 Patienten (15,7 %). Insgesamt wurde bei 4 Patienten (5,7 %) ein ISS von 9 bis 15 Punkten („moderate trauma“) und bei 16 Patienten (22,9 %) ein ISS von 16 bis 24 Punkten [„severe trauma“ 2015 vs. 2017 vs. 2019: *n* = 3 (15,0 %) vs. *n* = 8 (28,6 %) vs. *n* = 5 (21,7 %)]. Der mediane ISS-Punktwert in den einzelnen NACA-Kategorien stieg kontinuierlich an (Zusatzmaterial online: Abb. S1).

**Abb. 5:**
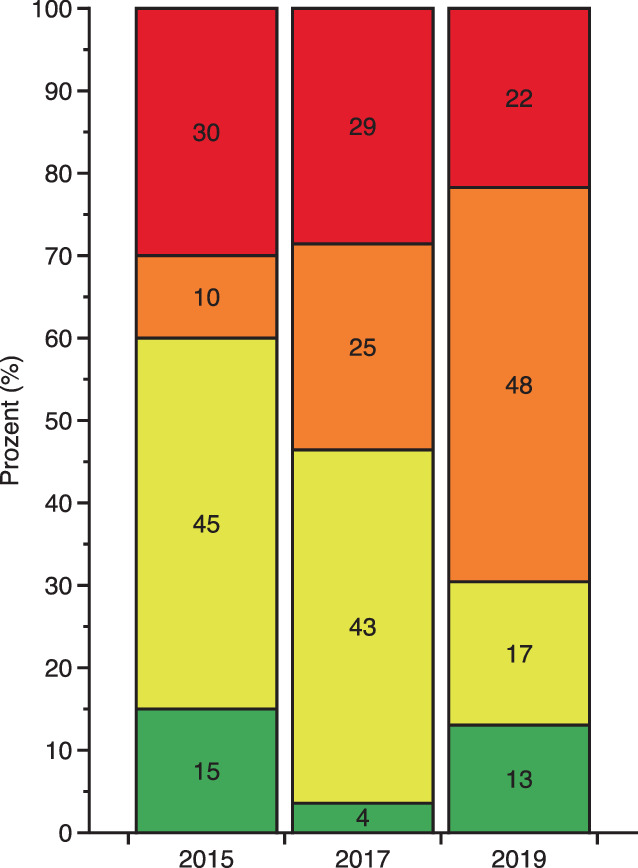
Verteilung der im überregionalen Traumazentrum zur Aufnahme gekommenen 71 Patienten mit penetrierenden gewaltassoziierten Verletzungen anhand der Kategorien des 5‑stufigen Manchester Triage System (MTS) in den Jahren 2015, 2017 und 2019. Patienten mit der MTS-Dringlichkeit „rot“ sind unmittelbar vital bedroht, absteigend nach Dringlichkeit und auch Zuordnung einer Zeit, bis zu der ein Arztkontakt stattgefunden haben soll, sind dann die Kategorien „orange“, „gelb“, „grün“ und „blau“

Betrachtet man die Manchester-Triage-System(MTS)-Kategorie orange/rot, so nahm der Anteil als kritisch eingeschätzter Patienten im Lauf der Jahre zu (2015 vs. 2017 vs. 2019: *n* = 8 (40,0 %) vs. *n* = 15 (53,6 %) vs. *n* = 16 (69,6 %)) (Abb. 5). Auch korrelierte die notärztlich prähospital eingeschätzte NACA-Kategorie mit der innerklinisch pflegegestützt eingeschätzten MTS-Kategorie (r^2^ = 0,67243, Zusatzmaterial online: Abb. S2).

**Abb. 6:**
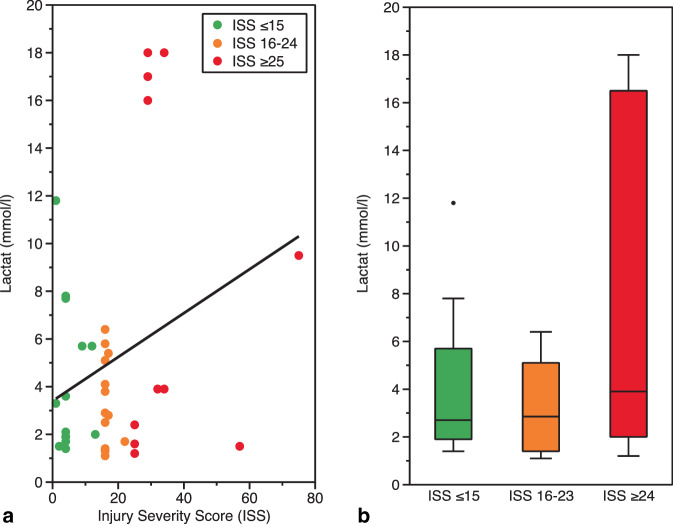
Verteilung der im überregionalen Traumazentrum zur Aufnahme gekommenen 71 Patienten mit penetrierenden gewaltassoziierten Verletzungen mit Darstellung (**a**) der Korrelation zwischen dem Lactatwert (in Millimol/Liter) und der Verletzungsschwere (Injury Severity Score, ISS) und (**b**) des Anstiegs des medianen Lactatwerts (Whisker-Plot) in Abhängigkeit von leicht (ISS ≤ 15), mittelschwer (ISS 16–23) und schwer verletzten Patienten (ISS ≥ 24)

Die Anzahl der Patienten, die pro untersuchtem Jahrgang über den Schockraum aufgenommen wurden (2015 vs. 2017 vs. 2019: *n* = 7 (35,0 %) vs. *n* = 9 (32,1 %) vs. *n* = 10 (43,5 %)), und die Anzahl der nach der Erstversorgung unmittelbar einer operativen Versorgung zugeführten Patienten (2015 vs. 2017 vs. 2019: *n* = 6 (7,4 %) vs. *n* = 8 (8,6 %) vs. *n* = 9 (9,8 %)) nahmen über die Jahre zu. Von den Patienten, die einer operativen Versorgung zugeführt worden sind, wurden 7 Patienten explorativ laparotomiert oder laparoskopiert (2015 *n* = 2; 2017: *n* = 3; 2019: *n* = 2). Bei einem Patienten war in 2017 wegen einer Verletzung des rechten Ventrikels intraoperativ der Einsatz einer Herz-Lungen-Maschine erforderlich. Der Anteil der Patienten mit positivem Alkoholnachweis in der Laborabnahme war über die Jahre zunehmend (2015 vs. 2017 vs. 2019: *n* = 2 (10,0 %) vs. *n* = 6 (21,4 %) vs. *n* = 10 (43,5 %)). Bei den 39 Patienten, bei denen eine Lactatbestimmung durchgeführt wurde, lässt sich ermitteln, dass ein normertiger Lactatwert (< 2,3 mmol/l) nicht mit der Verletzungsschwere korreliert. Lactatwerte ≥ 8 mmol/l fanden sich nur bei Patienten mit einem hohen ISS-Punktwert (Abb. 6).

Die Konversionsrate (stationäre Aufnahmerate) betrug 50,7 % (36 von 71 Patienten). Die Liegedauer der stationär aufgenommenen Patienten nahm über die Jahre ab (2015 vs. 2017 vs. 2019: 8,7 vs. 7,2 vs. 6,1 Tage). Die innerklinische Letalität betrug 1,4 % (*n* = 1).

## Diskussion

Die vorliegende retrospektive GewPen-Studie liefert erstmals Daten zu penetrierenden, gewaltassoziierten Verletzungen in 3 Einzeljahrgängen und insgesamt einen Zeitraum von 5 Jahren für einen großen städtischen Rettungsdienstbereich. Haupteinsatzlokalisationen und damit Ereignisschwerpunkt penetrierender und gewaltassoziierter Verletzungen waren in allen 3 untersuchten Einzeljahrgängen die Altstadt, die Stadtmitte und „Oberbilk“ als unmittelbar angrenzender Stadtteil. Eine besonders hohe Einsatzhäufigkeit fand sich in den Nächten von Samstag auf Sonntag zwischen 20.00 und 04.00 Uhr. Die am häufigsten betroffene Altersgruppe war zwischen 15 und 34 Jahre alt. Führende Tatwaffen bzw. -gegenstände waren in über 50 % der Fälle Messer, gefolgt von abgeschlagenen Glasflaschen und Scherben. Die im transsektoralen Ansatz erfassten innerklinischen Versorgungsdaten des ÜTZ wiesen auf Zunahmen der Verletzungsschwere und eines positiven Alkoholnachweises sowie auf eine häufigere unmittelbare operative Versorgung der verletzten Patienten über die Jahre hin. Die gewonnenen Erkenntnisse können einen Beitrag zur Entwicklung einer Versorgungsstrategie und Prävention leisten.

Im internationalen Vergleich sind penetrierende Verletzungen in Deutschland gegenüber stumpfen Traumata viel seltener: Für die Vereinigten Staaten (USA) werden im Vergleich zu anderen europäischen Ländern deutlich höhere Inzidenzen penetrierender Verletzungen angegeben: Während in den USA penetrierende Verletzungen einen Anteil von 20–45 % aller Verletzungen ausmachen, ist dieser Anteil für die Niederlande mit 3–4 % und für die Schweiz mit nur 0,2 % deutlich geringer [9]. Das nationale TraumaRegister der Deutschen Gesellschaft für Unfallchirurgie (TR-DGU) weist für einen 10-jährigen Zeitraum (2012–2021) anhand von 313.461 mit 95,9 % überwiegend einen stumpfen und mit nur 4,1 % in einem geringen Anteil penetrierende Verletzungen nach [6]. Die Datenanalysen des TR-DGU der einzelnen Jahrgänge 2012 (*n* = 28.805, [8]) und 2021 (*n* = 28.580 [6]) unterscheiden sich dabei hinsichtlich des Anteils penetrierender Verletzungen mit 5,1 und 4,2 % wenig, auch wenn diese Reduktion aufgrund der hohen Anzahl an eingeschlossenen Patienten eine statistische Signifikanz erreicht. In der deutlich kleineren Patientenkohorte der vorliegenden retrospektiven GewPen-Studie über einen 5‑jährigen Zeitraum (2015–2019) konnte die Reduktion penetrierender Traumata nicht nachgewiesen werden.

In einer weiteren Analyse des TR-DGU mit 9575 Patienten im Zeitraum von 2009–2018 konnte nachgewiesen werden, dass das Alter von Patienten mit penetrierenden Verletzungen stark von der Tatwaffe abhängig ist [3]. Während Patienten mit Schussverletzungen (*n* = 1123, ISS 23 ± 15, männlich 89 %) ein durchschnittliches Alter von 53 ± 21 Jahre aufwiesen, betrug das Alter bei Messerstichverletzungen (*n* = 4333, ISS 14 ± 10, männlich 84 %) 39 ± 17 Jahre und bei anderen penetrierenden Ursachen (z. B. Verkehrsunfällen mit Kraftfahrzeugen, Motor- und Fahrrädern, Fußgängern und Stürze, *n* = 4119, ISS 18 ± 13, männlich 76 %) 47 ± 21 Jahre [3]. Im Einklang zu dieser Analyse des TR-DGU wiesen die Patienten der GewPen-Studie mit einem hohen Anteil an Messerstichverletzungen ein ähnliches Alter bei gleichzeitig ebenfalls deutlichem Überwiegen des männlichen Geschlechts auf. Auch eine weitere Analyse aus dem TR-DGU aus dem Jahr 2014 [2] und eine retrospektive Untersuchung aus Finnland aus dem gleichen Jahr [16] beschreiben ähnliche epidemiologische Charakteristika. Die demografische Zusammensetzung des Patientenkollektivs der GewPen-Studie könnte darüber hinaus maßgeblich dadurch geprägt sein, dass die meisten Ereignisse an Event-Brennpunkten in der Düsseldorfer Altstadt (29 %), der Stadtmitte (18 %) und einem angrenzen Stadtteil (9 %) stattfanden. Hier findet sich ausgesprochen viel Gastronomie („die längste Theke der Welt“) als Anzugspunkt der in der retrospektiven Kohortenanalyse am häufigsten betroffenen Patientengruppe im Alter zwischen 14 und 34 Jahren. Dieses Studienergebnis und die Erläuterung des gastronomischen Umfelds zum Ereignisschwerpunkt erklärt dann auch konkludent, dass die meisten Einsätze (43 %) am Wochenende (Samstag und Sonntag) bzw. in 55 % abends bzw. nachts zwischen 20.00 und 04.00 Uhr stattfanden. Auch in der bereits angeführten Untersuchung aus dem TR-DGU [2] wurden penetrierende Traumata gehäuft am Wochenende und in der zweiten Tageshälfte erfasst. Auch die erwähnte Untersuchung aus Finnland scheint diese Beobachtung zum tageszeitlichen Patientenaufkommen zu bestätigen [16].

Tatwaffen bzw. -gegenstände waren in der vorliegenden GewPen-Studie in mehr als der Hälfte der Fälle Messer (56 %), gefolgt von abgeschlagenen Glasflaschen (18 %) und Scherben (6 %). Dieses Ergebnis steht im Einklang mit anderen Arbeiten, in denen die Mehrzahl penetrierender Verletzungen durch Messer verursacht wurde [18, 29].

Bedrohungen mit Messern wurden in die vorliegende Studie nicht aufgenommen, hier können abweichende Zahlen im Vergleich zu Kriminalitätsstatistiken vorliegen. Hervorzuheben ist allerdings im Jahr 2017 ein Amoklauf im untersuchten Rettungsdienstbereich, bei dem 5‑mal eine Axt als Tatwaffe eingesetzt wurde [23].

Erwartungsgemäß und im Einklang mit der nationalen Literatur wurden penetrierende Verletzungen durch Schusswaffen in der GewPen-Studie nur in 3 % der Fälle dokumentiert und stellen damit einen sehr geringen Anteil dar. Unterschiede in der Verteilung Stich- vs. Schusswaffen ergeben sich durch die Einschlusskriterien der zugrunde liegenden Studien: Im TR-DGU wird jede Verletzung mittels des Abbreviated Injury Score (AIS) einem genauen Punktwert zugeordnet, aber erst ab einem AIS > 1 wird diese Verletzung im TR-DGU berücksichtigt, während in der GewPen-Studie alle gewaltassoziierten penetrierenden Verletzungen erfasst wurden. Die unterschiedlichen Einschlusskriterien erklären einerseits den deutlich höheren Anteil von Stichwaffen im Verletzungsspektrum der GewPen-Studie als auch andererseits die mit 62 % vorwiegende Erfassung von Rettungsdiensteinsätzen mit geringer Verletzungsschwere (NACA 1–2). Obwohl die Verletzungsschwere gemäß ISS mit dem Schweregrad mittels NACA-Kategorie zunahm, muss einschränkend konstatiert werden, dass der NACA-Score methodisch möglicherweise ungeeignet ist, um die Erkrankungs‑/Verletzungsschwere von Patientenkollektiven sinnvoll abzuschätzen. Beispielsweise ist der NACA-Score abhängig von der Einsatzerfahrung des einschätzenden Notarztes [17]. Um aber überhaupt eine Vergleichbarkeit für die präklinische Versorgung zu erhalten, wurde sich dennoch für die Nutzung des NACA-Score entschieden. Lässt man also diese Limitation einmal außer Acht, so wies jeder 5. Patient eine akute Lebensgefahr nach rettungsdienstlicher Einschätzung auf. Interessant in diesem Zusammenhang ist die über die Jahre signifikante Steigerung von primären Notarzteinsätzen bzw. Notarztnachforderungen. Die Inzidenz von Todesfällen noch an der Einsatzstelle von 1 % aufgrund penetrierender Verletzungen erscheint vor dem Hintergrund der internationalen Literatur mit 15 % niedrig [25].

Im internationalen Vergleich finden sich eine Vielzahl an Studien, die einen deutlich höheren Anteil an Schussverletzungen beinhalten (schwedisches Traumaregister Stich- vs. Schusswaffe: 55,5 vs. 37 %) [12]. Dabei ist das Gesetz bezüglich des Führens von Messern in Schweden sogar noch etwas strenger als das entsprechende Gesetz in Deutschland: In Schweden ist das Tragen von Messern im öffentlichen Raum seit 2022 verboten, hier gibt es hohe Geldstrafen bis hin zu einer Haftstrafe. In Deutschland ist dies von der Art des Messers und von der Länge der Klinge abhängig. Auch US-amerikanische Untersuchungen weisen einen deutlich höheren Anteil an Schusswaffen auf (Stich- vs. Schusswaffe: 64 vs. 36 %) [9], und Schussverletzungen nehmen den 3. Platz aller Todesursachen mit einem Anteil von 15 % in den USA ein [5]. Diesen gravierenden Unterschieden liegen vermutlich die deutlich restriktiveren Schusswaffengesetze in Deutschland und die damit verbundene geringere Verfügbarkeit zugrunde: Analysen weisen eine klare Korrelation zwischen in einem Land bestehender restriktiver Gesetzgebung und der Anzahl an schusswaffenassoziierten Todesfällen auf [10, 21, 22].

Das in der GewPen-Studie nachgewiesene Verletzungsmuster mit vorwiegend Verletzungen der oberen Extremität im Sinne von Abwehrverletzungen und des Kopfes/Halses als Folge von Stichverletzungen ist typisch für körperliche Auseinandersetzungen. Die weiteren betroffenen Regionen Thorax und Abdomen sind mit 30 % deutlich seltener vertreten als in anderen Patientenkollektiven mit bis zu 80 % [3]. Dass in 16 % Verletzungen von mehr als einer Körperregion dokumentiert wurden, spricht für die Aggressivität dieser Auseinandersetzungen.

Notfallmedizinische Versorgungskonzepte sind für penetrierende Verletzungen auch in Deutschland eingehend beschrieben worden [13]. Dabei ist zu beachten, dass nach den Ergebnissen der GewPen-Studie nur jeder 4. prähospital behandelte Patient einen periphervenösen Zugang erhielt. Diese Beobachtung spricht für das insgesamt durch eine niedrige Verletzungsschwere gekennzeichnete Patientenkollektiv. Dabei stehen grundsätzlich für die Versorgung von penetrierenden Verletzungen spezifische notfallmedizinische Maßnahmen (z. B. Tourniquet, Pleuraraumentlastung, Atemwegssicherung, Chest-Seal-Schnellverband) oder Pharmaka (z. B. Tranexamsäure, Hämostyptikum) zur Verfügung. Im Nachgang zu weltweit beachteten Terroranschlägen wurden auch im Rettungsdienstbereich Düsseldorf die Aspekte Sichtung, Vorhaltung von spezifisch adaptiertem Equipment auf Rettungsmitteln und fortlaufende Schulungsmaßnahmen des Personals (z. B. Skillstrainings (Tourniquetanlage [14, 20]), szenarienbasierte Übungen, Tactical Combat Casualty Care (TCCC [4, 28])) etabliert. Hierfür eignen sich Skilltrainings an speziellen Trainingspuppen, gegenseitige Anlage von Tourniquets bei Teilnehmern einer Fortbildung, Anlage von Thoraxdrainagen (z. B. am Schweinemodell), spezielle Verbandtechniken (z. B. Olaes-Bandage) bei Verletzungen am Hals oder im Bereich der Leiste, Anwendung von Hämostyptika unter Zuhilfenahme manueller Kompression und Druckverbänden, kontinuierliche beispielweise jährliche Schulungen (z. B. im Rahmen der verpflichtenden 30-h-Rettungsdienst-Fortbildung und in Notarzttrainings). Die niedrige Häufigkeit des Einsatzes mit speziellen notfallmedizinischen Interventionen bei gewaltassoziierten penetrierenden Verletzungen zeigt die Notwendigkeit für nachhaltige Trainingskonzepte, damit entsprechende Techniken beim wirklichen Ernstfall sowohl im Rettungs- als auch im Notarztdienst sicher durchgeführt werden können.

Im transsektoralen Forschungsansatz konnten im ÜTZ 27 % der Patienten der Gesamtkohorte weiterevaluiert werden. Obwohl diese Patienten aufgrund eines Dispositionsbias nicht als sicher repräsentativ für das Gesamtkollektiv gewertet werden können, lassen sich zumindest für Maximalversorger entsprechende Rückschlüsse ziehen: Rund die Hälfte der ÜTZ-Patienten wies eine geringe Verletzungsschwere mit ISS < 9 Punkten auf. Dieses Ergebnis korrespondiert zur rettungsdienstlichen und notärztlichen Einschätzung der Gesamtkohorte. Andererseits lag bei der anderen Hälfte der Patienten eine relevante Verletzungsschwere vor (ISS > 9 Punkte). Die innerklinische Ersteinschätzung weist hierbei eine Vergleichbarkeit mit einem Anteil an MTS-Kategorien „orange“ oder „rot“ eingeschätzten Patienten von 2015 mit 40 % bis 2019 mit 70 % auf. Vergleichbar zum Anstieg der kritischen MTS-Kategorien stieg die pro Jahr über den Schockraum aufgenommene Anzahl an Patienten an. Gleichermaßen nahm der Anteil an Patienten zu, die unmittelbar nach der Erstversorgung in der Notaufnahme einer operativen Versorgung zugeführt werden musste. Aus innerklinischer Perspektive zeigen sich also Zunahmen der Verletzungsschwere und des Anteils vital bedrohter Patienten. Dabei scheint Lactat ein wichtiges diagnostisches Instrument zu sein, da Patienten mit schwereren Verletzungen ein erhöhtes Lactat aufweisen [19]. Interessanterweise stieg über die Jahre der Anteil alkoholisierter Verletzter auf über 40 % an. Diese Ergebnisse sind gut vergleichbar mit Studienergebnissen aus Finnland, die zeigen, dass Patienten mit penetrierenden Traumata in 57 % alkoholisiert waren [16].

Aus den vorliegenden Ergebnissen lassen sich einige einsatztaktische Vorschläge und Präventionsstrategien ableiten, die die Häufigkeit und Auswirkungen von penetrierenden Verletzungen möglicherweise zukünftig weiterreduzieren könnten: Zwischenzeitlich wurden temporäre Waffenverbotszonen für die Altstadt und den Hauptbahnhof Düsseldorf bereits umgesetzt. Darüber hinaus kann ein glasfreier Ausschank mit Plastikgläsern und -flaschen die Möglichkeit des Einsatzes von Scherben und zweckentfremdeten Flaschenhälsen reduzieren, wie es bereits bei Großveranstaltungen (z. B. Karneval) praktiziert wird. Eine Ausweitung des glasfreien Ausschanks und eine Steuerung des Alkoholkonsums können daher diskutiert werden. Möglicherweise würde dies aber nur zu einer Verlagerung der Menschenströme in Regionen ohne eine solche Verbotszone führen. Ohne sozioökonomische Faktoren in der vorliegenden Untersuchung erfasst zu haben, können als weitere präventive Maßnahmen sozialarbeitergestützte Maßnahmen im Hinblick auf Obdachlosigkeit und Drogenabhängigkeit diskutiert werden.

### Limitationen

Die GewPen-Studie ist durch das retrospektive Studiendesign und die Fallzahl limitiert. Jedoch ermöglicht die Analyse der vorliegenden Daten in dem gewählten Setting erstmals belastbare Ergebnisse für den Rettungsdienstbereich zu penetrierenden gewaltassoziierten Verletzungen. Die Erkenntnisse zur weiteren innerklinischen Versorgung und insbesondere hinsichtlich des Behandlungsergebnisses sind durch eine Beschränkung auf das ÜTZ limitiert. Aufgrund des Studiendesigns und aus Datenschutzgründen konnten aber keine Patienten erfasst werden, die in einem der anderen der 9 Krankenhäuser des Rettungsdienstbereiches zur Aufnahme kamen. Vor diesem Hintergrund könnte an dieser Stelle ein Dispositionsbias vorliegen, da möglicherweise nur die schwerer verletzten Patienten der Versorgung im Maximalversorger zugeführt wurden. Von einer Generalisierung der lokalen Ergebnisse auf das gesamte Stadtgebiet muss daher abgesehen werden. Die vorliegende Analyse scheint aber für das ÜTZ reliable Ergebnisse zu liefern.

## Fazit für die Praxis

- Insgesamt handelt es sich bei gewaltassoziierten penetrierenden Traumata um seltene Verletzungen in der prähospitalen und innerklinischen Einsatzrealität der untersuchten Metropolregion.
- Besondere Vorbereitungen im Sinne der Ausstattung von Rettungsmitteln als auch in der Schulung des eingesetzten Personals sind notwendig.
- Zukünftige Ausbildung des ärztlichen- und nichtärztlichen Rettungsdienstpersonals sowie Klinikpersonals und entsprechende Versorgungskonzepte von gewaltassoziierten penetrierenden Traumata sind für die Präklinik und Notaufnahmen von besonderer Relevanz.
- Aus den vorliegenden Ergebnissen lassen sich einige einsatztaktische Vorschläge und Präventionsstrategien ableiten, die die Häufigkeit und Auswirkungen von penetrierenden Verletzungen möglicherweise zukünftig weiterreduzieren könnten.
- Eine Ausweitung des glasfreien Ausschanks mit Plastikbechern und -flaschen kann die Möglichkeit des Einsatzes von Scherben und zweckentfremdeten Flaschenhälsen reduzieren, wie es bereits bei Großveranstaltungen (z. B. Karneval) praktiziert wird.
- Eine Steuerung des Alkoholkonsums könnte diskutiert werden. Möglicherweise würde dies aber nur zu einer Verlagerung der Menschenströme in Regionen ohne eine solche Verbotszone führen.

### Supplementary Information

Zunahmen der Verletzungsschwere gemäß ISS in den einzelnen NACA-Kategorien und Korrelation des NACA-Scores und der ersteingeschätzten MTS-Kategorie
