# Supplementary material for: Development of violence-associated penetrating trauma in the Düsseldorf metropolitan region over a 5-year period (GewPen study)
Source: Anaesthesiologie. 2024 May 22;73(7):444–53. [Article in German] doi: 10.1007/s00101-024-01420-6 (PMC11222214; doi:10.1007/s00101-024-01420-6)
Supplement: Supplementary file 1 — Zunahmen der Verletzungsschwere gemäß ISS in den einzelnen NACA-Kategorien und Korrelation des NACA-Scores und der ersteingeschätzten MTS-Kategorie [file 101_2024_1420_MOESM1_ESM.pdf]

Online-Zusatzmaterial

Entwicklung gewalt-assoziierter penetrierender Traumata in der Metropolregion Düsseldorf über einen fünfjährigen Zeitraum (GewPen-Studie)

Jutta Schürmann, Mark Michael, Olaf Picker, Dan Bieler, Kalle Heitkötter, Thomas Tremmel, Bernd Schnäbelin, Michael Bernhard

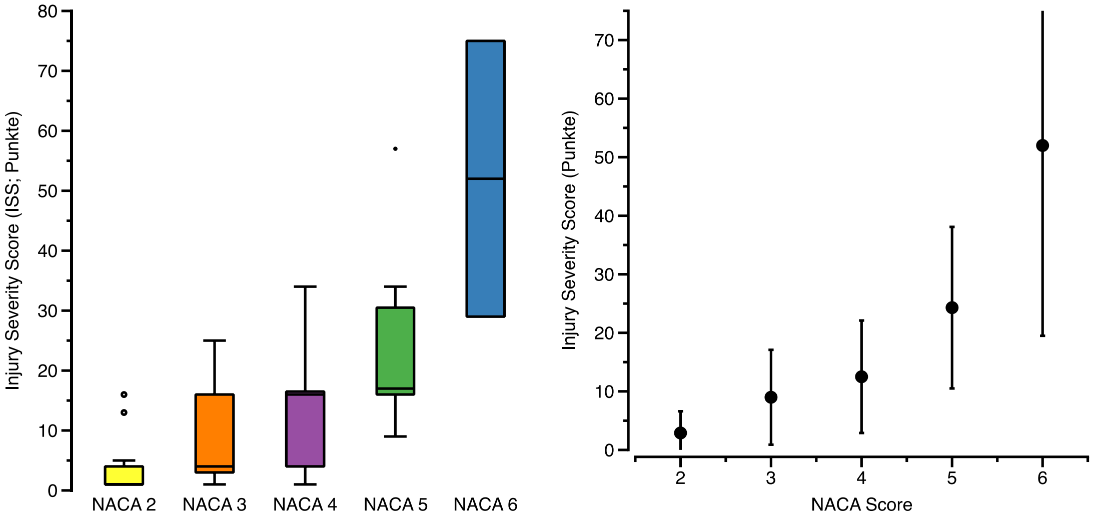

Abb. S1 Zunahmen der Verletzungsschwere gemäß Injury Severity Score (ISS) in den einzelnen NACA-Kategorien der 71 transsektoral untersuchten Patienten mit gewalt-assoziierten, penetrierenden Verletzungen: a) Whisker-Plot, b) Mittelwert ±Standardabweichung.

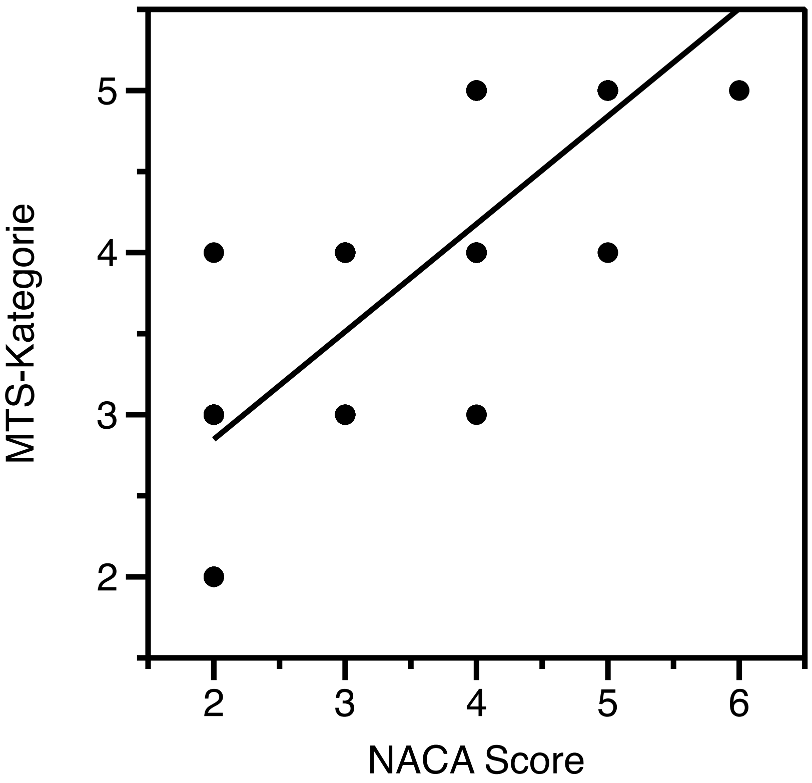

Abb. S2 Korrelation des NACA-Scores und der ersteingeschätzten MTS-Kategorie der 71 transsektoral untersuchten Patienten mit gewalt-assoziierten, penetrierenden Verletzungen (r^2=0,67243).
